# Glycoengineered Outer Membrane Vesicles: A Novel Platform for Bacterial Vaccines

**DOI:** 10.1038/srep24931

**Published:** 2016-04-22

**Authors:** Nancy L. Price, Guillaume Goyette-Desjardins, Harald Nothaft, Ezequiel Valguarnera, Christine M. Szymanski, Mariela Segura, Mario F. Feldman

**Affiliations:** 1 Department of Biological Sciences, University of Alberta Edmonton, Alberta T6G 2E9 Canada; 2 Laboratory of Immunology, Faculty of Veterinary Medicine, University of Montreal Saint-Hyacinthe, Quebec, J2S 2M2 Canada; 3 Department of Molecular Microbiology, Washington University School of Medicine, St Louis, MO 63110, USA

## Abstract

The World Health Organization has indicated that we are entering into a post-antibiotic era in which infections that were routinely and successfully treated with antibiotics can now be lethal due to the global dissemination of multidrug resistant strains. Conjugate vaccines are an effective way to create a long-lasting immune response against bacteria. However, these vaccines present many drawbacks such as slow development, high price, and batch-to-batch inconsistencies. Alternate approaches for vaccine development are urgently needed. Here we present a new vaccine consisting of glycoengineered outer membrane vesicles (geOMVs). This platform exploits the fact that the initial steps in the biosynthesis of most bacterial glycans are similar. Therefore, it is possible to easily engineer non-pathogenic *Escherichia coli* lab strains to produce geOMVs displaying the glycan of the pathogen of interest. In this work we demonstrate the versatility of this platform by showing the efficacy of geOMVs as vaccines against *Streptococcus pneumoniae* in mice, and against *Campylobacter jejuni* in chicken. This cost-effective platform could be employed to generate vaccines to prevent infections caused by a wide variety of microbial agents in human and animals.

Most successful current antibacterial vaccines are glycoconjugates, composed of cell surface carbohydrates chemically attached to an appropriate carrier protein. These are effective means to generate protective immune responses to prevent a wide range of diseases. One of the best examples is the conjugate vaccine against *Haemophilus influenzae* type b, which practically eliminated the disease caused by this bacterium in vast parts of the world. Other examples are the vaccines against *Streptococcus pneumoniae* and *Neisseria meningitidis*, both based on capsular polysaccharides^1^,^2^. However, the current technology to produce conjugate vaccines presents some drawbacks^3^,^4^,^5^. These require complex synthetic chemistry for obtaining, activating, and attaching the polysaccharides to protein carriers. The carbohydrates employed for the formulation of these vaccines are usually obtained from pathogenic organisms, which may constitute a hazard and may require higher levels of biosafety for production. Some of the glycans containing acid-labile sugars do not resist the chemical treatment required for their purification and crosslinking to proteins. Furthermore, the chemical crosslinking is often not reproducible, resulting in unreliable products with batch to batch variability. Novel approaches are needed to effectively prevent bacterial infections, especially in the context of the alarming increase of MDR bacterial strains.

Gram-negative bacteria are able to produce outer membrane vesicles (OMVs). OMVs are mainly composed of LPS, outer membrane and periplasmic proteins, and phospholipids^6^. OMVs are formed by blebbing of the outer membrane, although their biogenesis is poorly understood^7^. Due to their immunogenic properties, self-adjuvanticity, ability to be taken up by mammalian cells, and capacity for enhancement by recombinant engineering, OMVs are attractive candidates for vaccine delivery platform^8^. Within the last 25 years, vesicle based meningococcal vaccines (i.e., MenBvac^®^, MeNZB^®^, and BexSero^®^) have been successfully developed and employed within various countries^9^,^10^,^11^,^12^,^13^,^14^,^15^,^16^. Due to their success, other OMV vaccine candidates for various pathogenic bacteria including *Vibrio cholerae*^17^,^18^, *Bordetella pertussis*^19^, *Burkholderia pseudomallei*^20^,^21^, *Acinetobacter baumannii*^22^ and even Gram-positive bacteria, such as *Bacillus anthracis*^23^, have been tested. Such OMV based vaccines involve direct manipulations of large volumes of pathogenic bacteria and may pack unwanted bacterial toxins.

Pneumococcal disease kills more patients worldwide than any other vaccine-preventable disease. Annual worldwide statistics show that 1.6 million people die of pneumococcal disease^24^. The current vaccine (Prevnar 13^®^) protects against the 13 predominant serotypes in the USA, but serotypes that are important in other countries are not included in the vaccine. Furthermore, serotypes with lower prevalence often take over the niche left by the serotypes included in the vaccines^25^. Therefore effective vaccines for the remaining *S. pneumoniae* capsule serotypes are needed. *Campylobacter jejuni* is a natural commensal in all birds, including chickens. Humans are frequently infected with *C. jejuni* through the consumption of improperly cooked poultry. In most cases, infection only causes diarrhea, fever and abdominal pain. However, approximately, one in one thousand patients will develop a severe polyneuropathy known as Guillain-Barré syndrome (GBS)^26^. One way to control human infection by *C. jejuni* would be to reduce the load of the bacterium in chickens. Here we present an alternative platform for vaccines consisting of glycoengineered OMVs (geOMVs) derived from non-pathogenic engineered *Escherichia coli* strains expressing bacterial surface glycans encoded by the bacterial pathogenic organisms. As a proof of principle, we demonstrate the efficacy of geOMVs as vaccines for *S. pneumoniae* serotype *14,* and the common foodborne pathogen *C. jejuni*.

## Results

### Generation of geOMV displaying S. pneumoniae serotype 14 capsule (CPS14)

**Figure 1:**
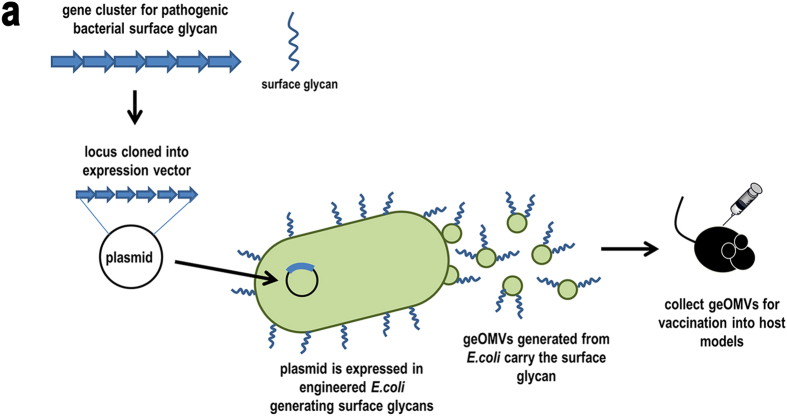
The geOMV platform. Genetic locus encoding glycan structure of interest is cloned into expression vector and introduced into an engineered *E. coli* strain which generates vesicles that display the glycan. The collected geOMVs can be directly utilized for immunizations.

**Figure 2:**
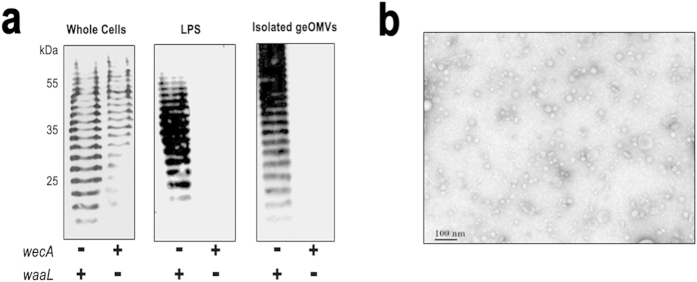
The *S. pneumoniae* serotype 14 capsule can be produced in *E. coli*. (**a**) Western blot analysis of CPS14 expressed in *E. coli* strains CLM37 (*wecA*-; *waaL*+) and CLM24 (*wecA*+; *waaL*-) show the production of the pneumococcal capsule in whole cell lysates, LPS extractions, and isolated OMVs. (**b**) TEM of isolated pneumococcal geOMVs.

The general strategy for the production of geOMV is represented in Fig. 1. In principle, our platform can be applied to any surface glycan, such as O antigens, capsules, exopolysaccharides, or glycans present in *N*- and *O*-glycoproteins. The initial steps in the biosynthesis of these glycans are common. These include the assembly of the glycan onto the undecaprenylpyrophosphate carrier and the flipping of the lipid-linked sugars across the inner membrane of Gram-negative or the cell membrane in Gram-positive bacteria. If these pathways are reconstituted in *E. coli*, the WaaL ligase transfers the glycan from the carrier to the lipid A, which is synthesized independently, and exported to the bacterial surface. OMVs displaying such antigens are then naturally produced. The biosynthetic pathways of type I capsular polysaccharides and O antigens only diverge once the polysaccharide has been translocated across the inner membrane (Supplementary Data, Fig. S1a). WaaL is not glycan-specific and can transfer a variety of sugars onto the lipid A core^27^. We predicted that expressing the *S. pneumoniae* capsule in an *E. coli* strain lacking its own O antigen would result in an LPS consisting of capsular polysaccharide attached to the lipid A core. To obtain such a strain, we constructed an inducible plasmid (pNLP80) which expresses the capsule synthesis *cps* locus and includes all essential glycosyltransferases, the flippase (Wzx), and the polymerase (Wzy) required for the synthesis of the *S. pneumoniae* serotype 14 capsule (CPS14). The expression of CPS14 was evaluated in two different *E. coli* mutant strains, CLM37^28^ and CLM24^29^ (Fig. 2a). CLM37 harbours a mutation which interrupts the initiator glycosyltransferase (*wecA)* for O antigen and Enterobacterial common antigen (ECA) synthesis^28^. In *Streptococcus* capsule synthesis the initiating glycosyltransferase is WchA, which transfers a glucose residue to the Und-PP carrier. WecA and WchA attach different sugars to the lipid and therefore would compete for the carrier. We reasoned that a *wecA* mutant strain, such as CLM37, in which competition for Und-PP is minimized, should produce more CPS14 than the wild-type strain. To demonstrate the attachment of the CPS14 to lipid A, we employed strain CLM24 (*waaL* mutant)^29^. In this strain the glycan remains attached to the lipid carrier and CPS14 should not be transferred to the lipid A. LPS extractions and OMV obtained from the two *E. coli* mutants show the presence of CPS14 in the WecA mutant but not in the WaaL ligase mutant strain, which indicates that CPS14 is attached to the lipid A core and directed to the vesicles in strains such as CLM37 that carry an intact WaaL (Fig. 2a). The signal detected in the WaaL mutant strain corresponds to UndPP-linked CPS14. The geOMVs were visualized by transmission electron microscopy (TEM) (Fig. 2b).

### geOMV displaying *S. pneumoniae* CPS14 raise specific antibodies and are effective in OPA assays

**Figure 3:**
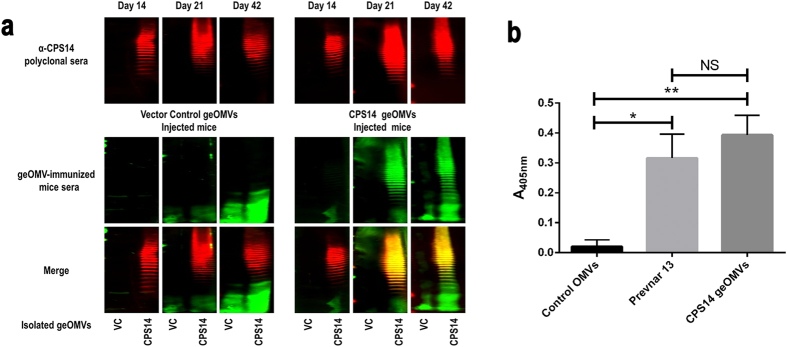
Sera from mice injected with geOMVs displaying *S. pneumoniae* serotype 14 capsule antigens cross react with the *S. pneumoniae* capsule. (**a**) Western blots of geOMVs from CLM37 strain with vector control (VC) or *S. pneumoniae* serotype 14 capsule (CPS14). The *S. pneumoniae* serotype 14 capsule was visualized by either the commercially available rabbit anti-capsule serotype 14 antibody (red) and the mice sera (green). The overlapping signals are shown in yellow (merged). (**b**) Pneumococcal geOMVs display the similar immunogenic response as the commercial available Prevnar 13 pneumococcal vaccine. Mice sera (n = 10) collected at day 42 were incubated in wells of ELISA plates seeded with whole cell *S. pneumoniae* serotype 14. Figure shows statistically significant differences between means for each group. (*p-value < 0.0001; **p-value < 0.0001; NS p-value 0.0298). Error bars are SD and P values were calculated using the unpaired t-test.

To determine whether geOMVs from CLM37 strain displaying the CPS14 would generate an immunogenic response in a murine model, we injected 2 μg of geOMVs per mouse (n = 10). Control groups immunized with either geOMVs lacking the capsule (empty geOMVs) or the commercially available Prevnar 13^®^, were included for comparison. Booster doses of geOMVs and Prevnar^®^ were administered on Days 14 and 28 and sera was collected weekly over a 42 day period. Collected sera of the immunized mice were assayed via Western blot analysis (Fig. 3a). Initial immunization with geOMVs did not elicit an IgG immunogenic response. However one week after the first booster dose (Day 21), high IgG titers in response to CPS14 were generated (Fig. 3a). The IgG response continued to be observed over the remaining course of the immunization schedule. To determine whether the immunogenic response was specific for serotype 14, we performed ELISAs with whole pneumococcal cells of serotypes 14 and 9 V (Supplementary Data, Fig. S1b,c). Sera from mice injected with geOMVs with CPS14 reacted only with *S. pneumoniae* serotype 14, thus demonstrating that the immunogenic response is specific for the glycoengineered serotype. Furthermore, at day 42, the levels of the IgG response of mice injected with pneumococcal geOMVs were comparable to the immunogenic response of mice injected with the commercially marketed pneumococcal vaccine Prevnar 13^®^ (Fig. 3b) which suggested that the level of the protective effect could also be similar.

**Figure 4:**
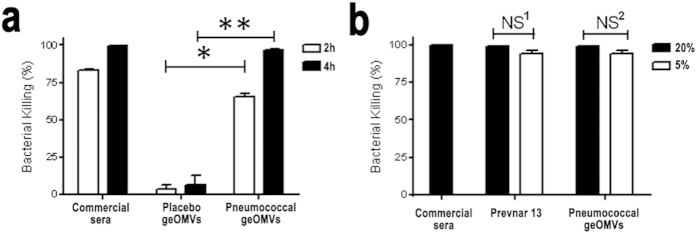
Bactericidal activity of sera from vaccinated mice against *S. pneumoniae* serotype 14. (**a**) Opsonophagocytosis assay (OPA) evaluation of the effect of the incubation time (2 h and 4 h) on *S. pneumoniae* type 14 killing in presence of either 20% (v/v) placebo or vaccinated mice sera. Statistically significant differences were found at 2 hours and at 4 hours between the placebo and the vaccinated mice sera (*p-value < 0.0001; **p-value < 0.0001). Plotted data are means of percentages and SD as error bars. Kruskal-Wallis (non-parametric) test was used to calculate p-values. (**b**) OPA evaluation of the effect of the serum concentration (5% and 20%) on *S. pneumoniae* type 14 killing at 4 h incubation in presence of sera from mice vaccinated with either a pneumococcal geOMVs or Prevnar 13 commercial vaccine, used for comparative purposes. Commercial rabbit anti-*S. pneumoniae* type 14 serum was used as positive control to validate OPA methodology. No statistically significant differences could be observed between different serum concentrations (NS^1^ p-value = 0.15; NS^2^ p-value = 0.21). P-values were calculated using Kruskal-Wallis test. No bacteria were found when using 20% serum concentration.

Opsonophagocytosis assays (OPA) are accepted as one the most reliable ways to evaluate the efficacy of pneumococcal vaccines^30^. OPA were performed to evaluate the efficacy of geOMVs against an *S. pneumoniae* serotype 14 infection. Day 42 sera obtained from the three groups of immunized mice (i.e., placebo geOMVs, pneumococcal geOMVs, and Prevnar 13^®^) were employed. OPA showed a significantly increased killing effect using pneumococcal geOMVs compared to placebo geOMVs (Fig. 4a), which suggests that isolated pneumococcal geOMVs elicit a protective effect *in vitro*. In addition, varying mouse serum concentrations (5% and 20%) showed a similar killing effect between pneumococcal geOMVs and the Prevnar 13 pneumococcal vaccine (Fig. 4b), thus demonstrating that geOMVs have a similar protective effect when compared to the commercially available Prevnar 13^®^ vaccine.

### geOMV reduce chicken colonization by *C. jejuni*

**Figure 5:**
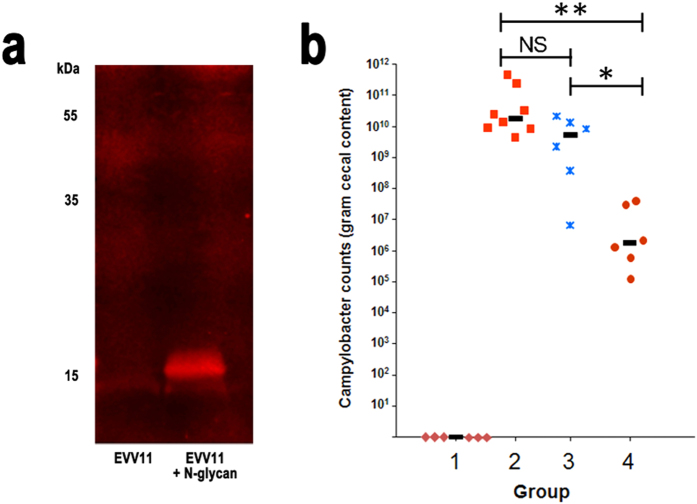
geOMVs displaying the *N*-heptasaccharide reduce *C. jejuni* colonization in chicken. (**a**) Western blots of *E. coli* EVV11 geOMVs displaying no oligosaccharides (lane 1) and the heptasaccharide *N*-glycan (lane 2). (**b**) *Campylobacter* colonization in chicken. Group 1, non-vaccinated, not challenged (background control); group 2, non-vaccinated, challenged (colonization control); group 3, vaccinated with empty OMVs and challenged; group 4, vaccinated with geOMVs expressing the *N*-glycan and challenged. Each data point represents cfu/gram cecal content for one bird. The median for each group is represented by a black bar. No significant difference in colonization could be observed between groups 2 and group 3 (NS p-value = 0.29). A statistically significant reduction in colonization was observed in group 4 birds that received the Cj-*N*-glycan presenting OMVs when compared to either group 3 (*p-value = 0.045) or group 2 (**p-value = 0.032). No *Campylobacter* cells were detected in group 1, the detection limit based on the experimental setup was 200 cfu/gram cecal content. P-values were calculated by the one-way ANOVA test.

We next evaluated the use of geOMVs to vaccinate chickens against *C. jejuni.* We introduced a plasmid (pACYCpglBmut) that expresses the *C. jejuni* heptasaccharide *N*-glycan into *E. coli* EVV11. This strain carries a mutation in Wzy and therefore does not polymerize the *C. jejuni* glycan as the wild-type strain does. We isolated geOMVs from this strain and analyzed them by Western-blot employing an *N*-glycan specific antibody. An immunoreactive signal migrating around 15 kDa that was absent in control OMVs clearly indicates that the *C. jejuni N*-glycan was displayed in geOMVs (Fig. 5a). To analyze the efficacy of the geOMVs containing the *C. jejuni N*-glycan, we tested the protective effect of these geOMVs in a *C. jejuni* chicken challenge model. Four groups of chickens (n = 8 for the positive control, n = 6 for the negative and experimental groups) were vaccinated orally with PBS, placebo geOMVs or *Campylobacter* geOMVs, and challenged with oral doses of PBS (negative control) or *C. jejuni* strain 81–176. Birds were monitored for 7 days before being euthanized and the amount of *C. jejuni* isolated from the cecum was determined for each bird. Birds that received PBS (Fig. 5b, group 2) and placebo geOMVs (Fig. 5b, group 3) showed similar levels of *C. jejuni* colonization after challenge. Chickens vaccinated with the *N*-glycan-containing geOMVs exhibited an almost 10^4^-fold reduction in *C. jejuni* colonization after challenge when compared to the naïve and placebo groups (Fig. 5b, group 4). In agreement with this data, the IgY levels of chicken that received the geOMV containing the glycan were higher than the ones that received the empty OMV (Fig. S1d).

## Discussion

Conjugate vaccines are an effective way to create a long-lasting IgG immune response. However, besides being expensive to produce, these vaccines present a series of drawbacks. Large volumes of pathogenic cells need to be cultured to obtain the polysaccharides and the chemical crosslinking of the sugars to the protein is complex, with large batch to batch variability. The development of novel conjugate vaccines is very slow. Diverse serotypes not included in Prevnar 13^®^ are prevalently being isolated in different geographical locations, and therefore novel technologies that can accelerate the development of multivalent vaccines are required. The generation of conjugate vaccines through exploitation of bacterial protein glycosylation systems is a very promising alternative, but not all the glycan chains are efficiently attached to proteins by the oligosaccharyltransferase PglB, which requires a HexNAc residue at the reducing end of the sugar chain^31^. PglL, the *N. meningitidis O*-oligosaccharyltransferase, can recognize a galactose but not glucose residue at the reducing end (Feldman, manuscript in preparation). Interestingly, about 90% of the *S. pneumoniae* strains contain glucose at the reducing end. Here we demonstrated that OMVs produced by glycoengineered *E. coli* expressing a glycan from unrelated bacterial pathogens, such as the CPS14 capsule from *S. pneumoniae* or the heptasaccharide derived from *N*-liked glycans from *C. jejuni*, can be effective in producing a significant immune response.

In the case of *S. pneumoniae*, geOMVs induced an immune response, as measured by serum IgG levels and efficacy in OPA tests, which was similar to the one generated by the most widely used commercial conjugate vaccine. In the future, a multivalent vaccine could be generated by mixing geOMVs carrying capsules from different serotypes. Furthermore, geOMVs could complement the current conjugate vaccines, especially for serotypes corresponding to glycan structures for which the conjugation has not been solved. Although the Western blots together with the specific antibody response to CPS14 and the OPA assays suggest that the right structure of the CPS14 has been displayed in the geOMVs, future work will confirm the exact structure attached to lipid A. In the case of the *C. jejuni* geOMV vaccine candidate, the 4 log reduction in chicken colonization is, to our knowledge, unprecedented for this microorganism. However, the power of the vaccine could be increased if, in addition to engineering *E. coli* to produce pathogenic bacterial surface glycans, the geOMVs were modified to express antigenic membrane proteins to be directed to the vesicles. Previous studies have already explored incorporating antigenic proteins from pathogenic species (*Neisseria, Streptococcus, Leishmania, Vibrio*, and *Yersinia*) into vesicles derived from laboratory *E. coli* and *Salmonella* strains^17^,^32^,^33^,^34^,^35^. Furthermore, OMV produced in *Salmonella* carrying pneumococcal protein antigens showed promise in murine models^36^. The geOMV platform would enable the glycans and proteins to synergistically increase the vaccine immunogenicity capacity. This can also be important for cases like in *S. pneumoniae*, in which a protein antigen could expand the protection to serotypes not included in the vaccines.

One of the main concerns with vesicle based vaccines is the safety issues as OMVs contain endotoxic LPS^37^. LPS lipid A has been shown to provoke severe/lethal inflammatory responses in the host^38^,^39^,^40^. The OMV vaccines employed in humans were derived from *Neisseria meningitidis*. Several studies have analyzed the effect of modifying the lipid A to abrogate its interactions to obtain OMVs with LPS preparations tailored for human vaccine development^41^,^42^,^43^. For example, *N. meningitidis* strains lacking LpxM or LpxL also render a lipid A with minimal toxicity^41^,^44^. It has been shown that OMVs produced in *E. coli* can also be detoxified through modifications of the lipid A^37^. These modifications can be carried out through the action of lipid A deacylases, such as PagL^45^. The overexpression of *B. pertussis* PagL resulted in OMVs with lower endotoxic activity compared to wild type *B. pertussis* OMVs. Monophosphorylated lipid A species recently became the first new Food and Drug Administration-approved adjuvant in several decades^46^. Therefore, it might be possible to generate geOMVs with a perfect balance between reduced toxicity and optimal adjuvanticity by generating strains containing modifications in the levels of lipid A acylation, phosphorylation, and/or other modifications.

## Material and Methods

### Animal ethics statement

All experiments involving mice were conducted in accordance with the guidelines and policies of the Canadian Council on Animal Care and the principles set forth in the Guide for the Care and Use of Laboratory Animals. The animal protocol was approved by the Animal Welfare Committee of the University of Montreal (protocol #RECH-1523). Studies involving chickens were carried out in accordance with the protocol approved by the Animal Care and Use Committee at the University of Alberta using a 35 day challenge protocol.

### Construction of pneumococcal glycan expression plasmid

The *cps* gene cluster responsible for the synthesis of *S. pneumoniae* capsule serotype 14, CPS14, (excluding the regulatory genes) was cloned via a 3-way ligation method. Two separate fragments of the CPS14 locus were amplified using a high fidelity polymerase (iProof™, BioRad). Fragment 1 amplified the *wchA-wchM* region using the forward primer (5′-ATAGAGCTCATGGATAAAAAAGGATTGGAAAT-3′) and reverse primer (5′-TTAAGAAATTCATCCTCATACAA-3′). Fragment 2 amplified the *wchM-wciY* region using the forward primer (5′-CTGGTCAACAAATATTAGAAAAA-3′) and reverse primer (5′ATACTCGAGATTCTTTCTGTAAACTCCAAAAA-3′). The underlined sequences denotes *Sac*I and *Xho*I restriction enzyme sites, respectively. The PCR fragments 1 and 2 were both designed to include the native *Hind*III site within the *wchM* gene, thus, after a *Hind*III RE digest, the two fragments were compatible and reconstituted the *wchM* gene after ligation. Both fragments were successfully amplified and digested with *Sac*I/*Hind*III (fragment 1) or *Hind*III/*Xho*I (fragment 2) and cloned into the plasmid vector pBBR1MCS-2, which was double digested with *Sac*I and *Xho*I, generating pBBR1MCS-CPS14. The cloned CPS14 locus was confirmed by restriction enzyme digests and sequencing. For proper expression of the CPS14 locus in *E. coli*, the CPS14 locus was subcloned into pWSK129. The *wchA-wciY* CPS14 fragment was cleaved from pBBR1MCS-CPS14 using *Sac*I and *Xho*I restriction enzymes. pWSK129 was digested with *Sac*I and *Sal*I restriction enzymes. As *Xho*I and *Sal*I generate compatible sticky ends, the *wchA-wciY* fragment was ligated into pWSK129. The resulting plasmid, named pNLP80 was introduced in *E. coli* strains.

### Isolation of geOMVs from *E. coli* expressing bacterial glycans

geOMVs were isolated following the protocol described by Haurat *et al*.^47^. Briefly, *E. coli* strains harbouring plasmids expressing genes involved in glycan synthesis (i.e., pNLP80, pEQ3, pACYCpglBmut) and the respective vector control plasmids were grown at 37 °C overnight with shaking in 50 mL of Luria-Bertani (LB) broth plus appropriate antibiotics. The following day, each culture was subcultured (1:100 dilution) into 1 L LB or Terrific Broth (TB) plus antibiotics and grown at 37 °C with shaking for 2 h (OD_600_ ~ 0.2). Inductions of glycans were performed as follows: IPTG was added to a final concentration of 0.1 mM to induce expression of *Streptococcus* CPS14 (pNLP80) while the *Campylobacter* heptasaccharide biosynthetic gene cluster on pACYCpglBmut is constitutively expressed. The cultures were grown for an additional 26 h at 37 °C with shaking. After incubation, cells were harvested by centrifugation at 23,000 × g for 15 min and the supernatant was collected. The supernantants were filtered twice through 0.44 μm and 0.2 μm filters to remove any residual intact cells. The filtered supernatants were ultracentrifuged at 210,000 × g for 3 h at 4 °C. The geOMV pellets were collected and resuspended in buffered saline (PBS).

### Analysis and quantification of geOMV

Presence of geOMVs containing *Streptococcus* CPS14 or *Campylobacter* heptasaccharide was confirmed by Western blotting. Quantification of geOMVs was performed by measurement of KDO using a modified protocol described by Lee and Tasi^48^. Commercially available anti-rabbit CPS14 antibody (Statens Serum Institut, Denmark) was used as positive control (1:1000 dilution) to visualize the presence of the pneumococcal capsule.

#### Transmission electron microscopy

Isolated geOMVs were absorbed onto carbon-coated copper membrane grids for 3 min. Excess geOMVs were blotted away from membrane grid and samples were negatively stained with 2% (w/v) uranyl acetate (3 min). The grids were analyzed for presence of geOMVs using a Morgagni (FEI) transmission electron microscope (Biological Sciences Microscopy Facility, University of Alberta).

#### Murine studies with Pneumococcal geOMVs

Three groups of BALB/c mice (n = 10 per group, female, 4–6 weeks old, obtained from Charles River, WA) were immunized intraperitoneally. The test group was injected with 2 μg of geOMVs isolated from *E. coli* CLM37 strain expressing CPS14 (pNLP80). A placebo control group received geOMVs isolated from *E. coli* CLM37 strain not expressing CPS14. The third group was injected with commercially available dose of Prevnar 13 (500 μL as supplied). Sera were collected weekly via tail bleeds over a 42 day period and booster doses were administered on Days 14 and 28. Final bleed (Day 42) was via cardiac puncture. The presence of antibodies against CPS14 was analyzed by Western-blot using Odyssey imaging systems (LI-COR Biosciences, USA). geOMVs preparations digested with proteinase K were analyzed using 1:500 dilutions of the mouse sera.

#### Whole cell ELISAs

*S. pneumoniae* (serotype 9 V and 14, Statens Serum Institut, Denmark) was grown overnight on blood agar plates at 37 °C with 5% CO_2_ aerobic conditions. The next day the cells were scraped from the agar plate, resuspended in 1× PBS, and heat inactivated by incubation at 60 °C for 2 h. The cells were diluted to OD_600_ ~ 0.6/mL in PBS with protease inhibitor and 100 μL were seeded into ELISA 96 well plates. The plates were incubated overnight at 4 °C. The following day, the wells were washed three times with 1× PBS before blocking with 2.5% skim milk for 2 h. The wells were washed three times with PBS and incubated with 100 μL of mouse sera (1:500 dilution) was added to each well and the plates were incubated at room temperature for 1 h. After incubation, the wells were washed again three times with PBS and 100 μL of IgG anti-mouse-alkaline phosphatase antibody was added to each well and incubated at room temperature for 1 h. After the secondary antibody incubation, the wells were washed again three times with PBS and 100 μL of p-nitrophenyl phosphate was added to each well and the plates were incubated at 37 °C for 1 h followed by reading the absorbance at 405 nm on a BioTek™ plate reader.

### Opsonophagocytosis Assay (OPA)

#### Blood collection

Blood was collected by intracardiac puncture from naïve female mice (Charles River, Wilmington, MA), treated with sodium heparin, then diluted to obtain 6.25 × 10^6^ leukocytes/mL in RPMI 1640 supplemented with 5% heat-inactivated fetal bovin serum, 10 mM HEPES, 2 mM L-glutamine and 50 μM 2-mercaptoethanol. All reagents were from Gibco (Invitrogen, Burlington, ON, Canada).

#### Bacterial suspension preparation

Isolated colonies on sheep blood agar plates of *S. pneumoniae* serotype 14 (Statens Serum Institut, Denmark) were inoculated in 5 ml of Todd-Hewitt Broth (THB) (Becton Dickinson, Mississauga, ON, Canada) and incubated for 16 h at 37 °C with 5% CO_2_. Working cultures were prepared by transferring 0.1 mL of 16 h-cultures into 10 mL of THB which was incubated for 5 h. Bacteria were washed 3 times and resuspended in PBS to obtain an OD_600_ value of 0.6, which corresponds to 2 × 10^8^ colony forming units (CFU)/mL. Final bacterial suspension was prepared in complete cell culture medium to obtain a concentration of 6.25 × 10^4^ CFU/mL. The number of CFU/mL in the final suspension was determined by plating samples onto Todd-Hewitt Agar (THA) using an Autoplate 4000 Automated Spiral Plater (Spiral Biotech, Norwood, MA).

#### Assay

Diluted whole blood (5 × 10^5^ total leukocytes) was mixed with 5 × 10^3^ CFU of *S. pneumoniae* (MOI of 0.01) and either 5% or 20% (v/v) of serum from control (placebo) or vaccinated mice in a microtube to a final volume of 0.2 mL. Microtubes were incubated for 2 h or 4 h at 37 °C with 5% CO_2_, with shaking. After incubation, viable bacterial counts were performed on THA using an Autoplate 4000 Automated Spiral Plater. Tubes with addition of naive mouse sera (5% or 20% v/v) or of commercial rabbit anti-*S. pneumoniae* type 14 serum (20% v/v) (Statens Serum Institut, Denmark), were used as negative and positive controls, respectively. The % of bacteria killed was determined using the following formula: % Bacteria killed = [1 − (bacteria recovered from sample tubes/ bacteria recovered from negative control tubes with naïve sera)] × 100. Final OPA conditions were selected based in several pre-trials using different incubation times and MOIs (data not shown). A more detailed procedure can be found in^49^.

#### Chicken vaccination and challenge

In general each group contained up to 8 leghorn birds (Poultry Research Facility, University of Alberta) that were randomly tested for the presence of *Campylobacter* on the day of hatch (Day 1) by plating cloacal swabs onto selective Karmali agar. In all cases no *Campylobacter* colonies were observed after 48 h of incubation under microaerobic conditions at 37 °C. Vaccination was performed by orally gavaging with 300 μL of PBS containing 500 ng of geOMVs on Days 7 and 21. Control groups were gavaged with 300 μL of PBS only.

Birds were challenged on day 28 by oral gavaging with either PBS (negative control) or with 300 μL PBS containing 10^2^ *C. jejuni* 81–176 cells. The challenge strain was prepared as follows: *C. jejuni* 81–176 was grown for 18 h on Mueller-Hinton (MH) agar and harvested with cold MH broth. Cells were washed twice with cold PBS and adjusted to an OD_600_ of 1.2 (OD_600_ of 1.2 equals 3 × 10^8^ cell/mL). Serial dilutions in PBS were performed dependent on the final amount of cells that were administered. For example: 3 × 10^2^ cells/mL [ = 1 × 10^2^ cells per 300 μL (=1 dose)]. Cells were maintained on ice until used. Birds were maintained for an additional 7 days after challenge and then euthanized. Ceca were removed and the contents were adjusted to 1 mg cecal content per 1 mL with sterile PBS. Aliquots of 10-fold serial dilutions (in PBS) of the cecal contents were plated onto selective Karmali agar. CFU were determined after incubation of the plates for 48 h under microaerobic conditions.

#### ELISA for *C. jejuni N*-glycan-specific antibodies

Blood samples were collected on Day 28 (vaccine response prior to challenge) and kept at 37 °C until a firm blood clot was formed. Samples were centrifuged (5 min, 18.000 × *g*, 4 °C) and the supernatants (sera) were transferred to fresh tubes. After addition of glycerol to a final concentration of 10%, sera were stored at −20 °C until further use. For ELISA coating, Campylobacter *N*-glycan compounds (Cj-N-glycan) and their chemical conjugation to the protein carrier bovine serum albumin (BSA) was performed as described^50^,^51^. Maxisorb plates (Thermo Fisher) were coated with 500 ng of BSA-Cj-N-glycan conjugate per well for 18 h at 4 °C. After removal of unbound antigen the plate was blocked for 1 h at room temperature with PBS-Tween, 5% skim milk. After discarding the blocking solution 100 μL of chicken sera diluted 1:10 in PBS-Tween, 1% skim milk was added to each well. Plates were incubated for 1 h at room temperature and washed 3 times for 5 min with PBS-Tween. After addition of 100 μL of 2^nd^ antibody solution (anti chicken IgY, diluted 1:500 in PBS-Tween, 1% skim milk) and incubation for 1 h at room temperature wells were washed 4-times for 5 min with 100 μL of PBS-Tween and developed using the 1-Step p-Nitrophenyl Phosphate (PNPP) assay following the instructions of the manufacturer (Thermo Fisher). Immuno-reactivity in each serum was determined after reading the plate at OD_405_ in a plate reader.

## Additional Information

**How to cite this article**: Price, N. L. *et al*. Glycoengineered Outer Membrane Vesicles: A Novel Platform for Bacterial Vaccines. *Sci. Rep.* **6**, 24931; doi: 10.1038/srep24931 (2016).

## Supplementary Material

Supplementary Information
