# Supplementary material for: Glycoengineered Outer Membrane Vesicles: A Novel Platform for Bacterial Vaccines
Source: Sci Rep. 2016 Apr 22;6:24931. doi: 10.1038/srep24931 (PMC4840304; doi:10.1038/srep24931)
Supplement: Supplementary Information [file srep24931-s1.pdf]

**Glycoengineered Outer Membrane Vesicles: A Novel Platform for Bacterial Vaccines**

Nancy L. Price[1], Guillaume Goyette-Desjardins[2], Harald Nothaft[1], Ezequiel Valguarnera[3], Christine M. Szymanski[1], Mariela Segura[2], and Mario F. Feldman[1,3]

[1]Department of Biological Sciences, University of Alberta
Edmonton, Alberta T6G 2E9 CANADA

[2]Laboratory of Immunology, Faculty of Veterinary Medicine, University of Montreal
Saint-Hyacinthe, Quebec, J2S 2M2 CANADA

[3]Department of Molecular Microbiology, Washington University School of Medicine. St Louis, MO. 63110, USA.

**Suplementary Information**

**Figure S1. Supplementary Data. (a)** The comparison of pneumococcal capsule synthesis and *E. coli* LPS/glycoprotein synthesis. **(b) & (c)** Sera from mice injected with geOMVs displaying pneumococcal serotype 14 capsule antigens is specific for *S. pneumoniae* serotype 14. Mouse sera from 10 animals (Day 21) were incubated in wells of ELISAs plates seeded with whole cell *S. pneumoniae* serotype 9V (b) or 14 (c). As a negative control, sera from 4 mice injected with empty control OMVs was tested on the same plate layout. Means from each group were compared and significant differences were found between control OMVs sera and CPS14 geOMVs sera in (c) (* p-value < 0.0001). No significant differences were found between groups in (b) (NS p-value = 0.45). Error bars are SD and p-values were calculated by the unpaired t-test. **(d)** ELISA data of the IgY response of chicken vaccinated with *Campylobacter* geOMVs. Each symbol represents data of one chicken and the median is indicated with a line. Animal sera (Day 28) was used at a dilution of 1:10.

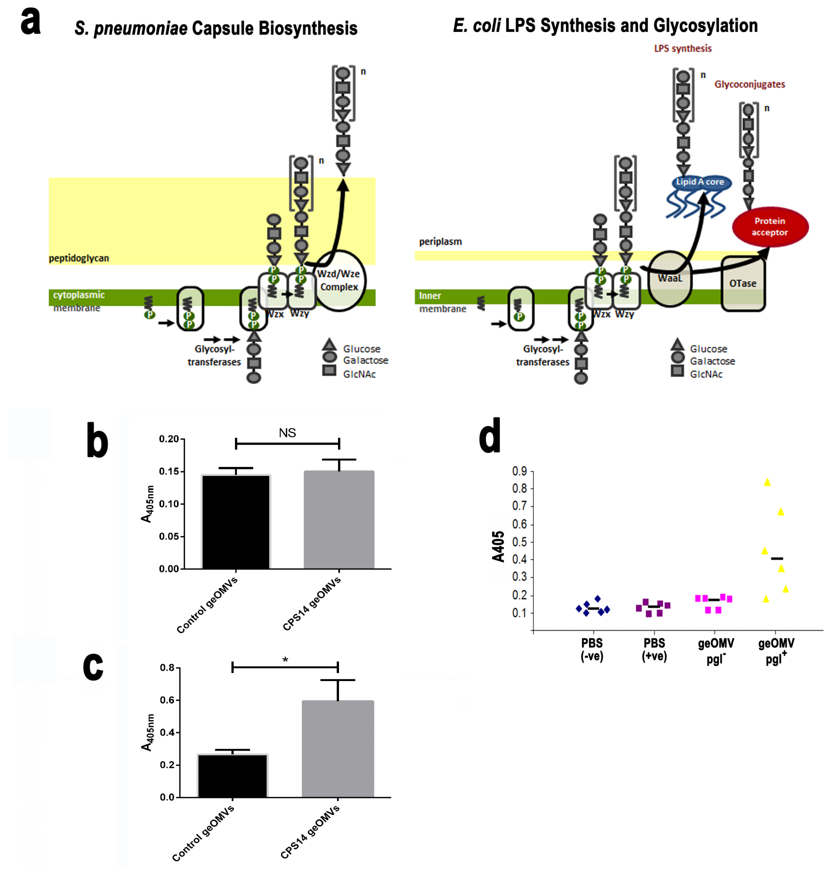
a
S. pneumoniae Capsule Biosynthesis
E. coli LPS Synthesis and Glycosylation
LPS synthesis
Glycoconjugates
n
peptidoglycan
cytoplasmic membrane
Wzd/Wze Complex
Wzx
Wzy
Glycosyl-transferases
Glucose
Galactose
GlcNAc
Lipid A core
Protein acceptor
periplasm
Inner membrane
WaaL
OTase
b
NS
$A_{405nm}$
0.20
0.15
0.10
0.05
0.00
Control geOMVs
CPS14 geOMVs
c
*
0.8
0.6
0.4
0.2
0.0
d
A405
0.9
0.8
0.7
0.6
0.5
0.4
0.3
0.2
0.1
PBS (-ve)
PBS (+ve)
geOMV pgl-
geOMV pgl+
